# A Systematic Review of the Effects of Interactive Telerehabilitation with Remote Monitoring and Guidance on Balance and Gait Performance in Older Adults and Individuals with Neurological Conditions

**DOI:** 10.3390/bioengineering11050460

**Published:** 2024-05-06

**Authors:** Catherine Park, Beom-Chan Lee

**Affiliations:** 1 Division of Digital Healthcare, Yonsei University, Wonju 26493, Republic of Korea; 2 Department of Health and Human Performance, University of Houston, Houston, TX 77204, USA; 3 Center for Neuromotor and Biomechanics Research, University of Houston, Houston, TX 77204, USA

**Keywords:** interactive telerehabilitation, monitoring, guidance, virtual reality, apps, videoconferencing, exergame

## Abstract

Recognizing the growing interests and benefits of technology-assisted interactive telerehabilitation in various populations, the aim of this review is to systematically review the effects of interactive telerehabilitation with remote monitoring and guidance for improving balance and gait performance in older adults and individuals with neurological conditions. The study protocol for this systematic review was registered with the international prospective register of systematic reviews (PROSPERO) with the unique identifier CRD42024509646. Studies written in English published from January 2014 to February 2024 in Web of Science, Pubmed, Scopus, and Google Scholar were examined. Of the 247 identified, 17 were selected after initial and eligibility screening, and their methodological quality was assessed with the National Institutes of Health Quality Assessment Tool for Observational Cohort and Cross-sectional Studies. All 17 studies demonstrated balance and gait performance improvement in older adults and in individuals with stroke, Parkinson’s disease, and multiple sclerosis following 4 or more weeks of interactive telerehabilitation via virtual reality, smartphone or tablet apps, or videoconferencing. The findings of this systematic review can inform the future design and implementation of interactive telerehabilitation technology and improve balance and gait training exercise regimens for older adults and individuals with neurological conditions.

## 1. Introduction

Across all age groups, maintaining stable balance and gait ensures mobility, movement coordination, and overall well-being [1]. Balance and gait impairments affect daily activities and quality of life, particularly by increasing the risk of falling and actual falls [2]. Indeed, falls are one of the most significant global health concerns, which can result in fractures, concussions, and even death [3]. Individuals with balance impairments often need balance rehabilitation to improve stability, reduce fall risk, and increase overall functional independence [4].

Systematic reviews of conventional balance rehabilitation regimens have documented balance and gait performance improvement in older adults [5] and individuals with stroke [6], Parkinson’s disease [7], traumatic brain injury [8], and multiple sclerosis [9]. Conventional balance rehabilitation regimens, however, often limit or prevent an individual’s full participation due to the unavailability of physical therapists, limited access to clinical facilities, reduced accountability, and cost [10,11,12]. Over time, an individual’s compliance and motivation may decrease in the absence of feedback [13] or having to perform the same exercise regimen [14]. 

Some studies have documented the recent effects of developing and accessing various telerehabilitation technology to improve balance and gait performance in different populations [15,16,17], such as virtual reality (VR), video games that use RGB-D cameras (e.g., Kinect), instrumented boards (e.g., Wii Balance Board), and motion sensors (e.g., inertial measurement units). These studies have concluded that VR or game-based telerehabilitation provides benefits including improved accessibility for individuals facing geographical barriers or with no transportation to traditional rehabilitation facilities; exercises tailored to individual needs and abilities; real-time feedback; more variety in exercise regimens; and increased exercise consistency.

Although two reviews have summarized the beneficial effects of videoconferencing interventions on telerehabilitation in older adults with musculoskeletal conditions [18] and individuals with stroke [19], there is no systematic review of the effects of telerehabilitation with remote monitoring and guidance to improve balance and gait performance in different populations. Indeed, remote monitoring and guidance allow for ongoing assessment, immediate feedback, adjustment of exercise intensity, timely interventions, and improved relationships between users and healthcare professionals, which can enhance the overall efficacy and success of the telerehabilitation program. Therefore, the aim of this review is to systematically review the effects of remote monitoring and guidance on telerehabilitation using VR, game-based systems, smartphone apps, and web-based videoconferencing for improving balance and gait performance in older adults and individuals with neurological conditions. This systematic review, in particular, includes studies that focus on the effects of interactive telerehabilitation on balance and gait performance in older adults and people with stroke, Parkinson’s disease, or multiple sclerosis, as well as the efficacy of interactive telerehabilitation over conventional rehabilitation (i.e., usual care, in-clinic, in-person, or in-home regimens).

## 2. Materials and Methods

### 2.1. Search Strategy 

This systematic review was conducted in accordance with the Preferred Reporting Items for Systematic Reviews and Meta-Analyses (PRISMA) criteria and guidelines [20]. Both authors determined appropriate databases, identified keywords, specified search terms, and developed the protocol for this systematic review. The Web of Science, PubMed, and SCOPUS databases and the Google Scholar search engine were used to search published articles. The search keywords, which was modified as needed, included all possible combinations of “telerehabilitation”; “telerehabilitation training”; “interactive telerehabilitation”; “balance”; “gait”; “remote*”; “bidirectional*”; “monitoring”; “guidance”; “older adults”; “stroke”; “Parkinson’s disease”; and “multiple sclerosis”. Manual searches of the reference lists used in previous systematic reviews of conventional balance and gait rehabilitation were also conducted. Searches were limited to data published between January 2014 and February 2024. Systematic, perspective, and narrative reviews, survey articles, and books and book chapters were excluded. The study protocol was registered with the international prospective register of systematic reviews (PROSPERO) with the unique identifier CRD42024509646.

### 2.2. Study Selection 

**Figure 1:**
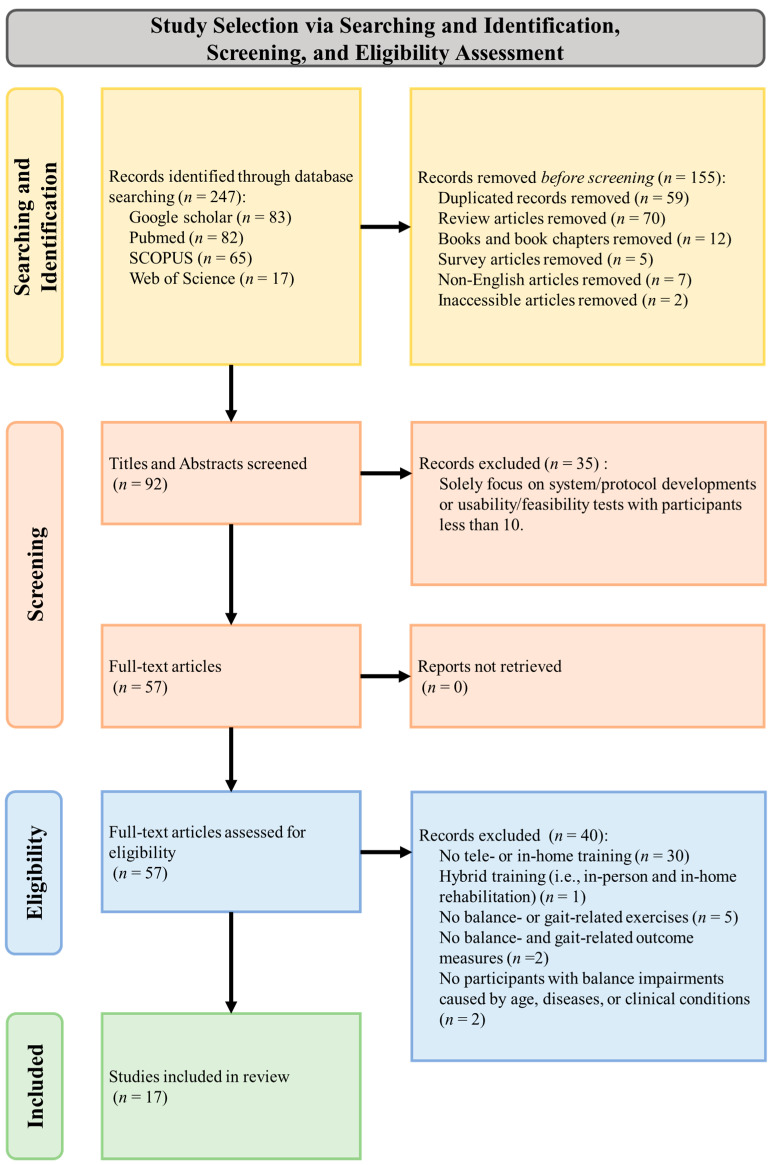
The PRISMA flow diagram illustrates the sequential process by which studies were identified, screened, assessed, and included in this review.

Both authors independently selected potential studies, and they then discussed and resolved any discrepancies through in-depth discussion and mutual agreement to determine the studies included in this systematic review. Figure 1 shows the flow diagram for study selection according to PRISMA criteria and guidelines. Studies were included if they were published in English, peer-reviewed, and full-text accessible; provided balance-related telerehabilitation exercises with remote monitoring or guidance (i.e., interactive telerehabilitation); provided remote interventions or remote tracking of progress; used technologies including VR or game-based systems, smartphone apps, or web-based videoconferencing; and included individuals with balance impairments (i.e., older adults and people with stroke, Parkinson’s disease, or multiple sclerosis).

Studies were excluded if they developed telerehabilitation technology only; evaluated telerehabilitation technology used in laboratory settings; developed experimental protocols; used fewer than 10 individuals to evaluate the feasibility and usability of telerehabilitation technology or telerehabilitation protocols with less than 10 individuals; used telerehabilitation without remote monitoring or guidance (i.e., non-interactive telerehabilitation); used hybrid telerehabilitation protocols (i.e., a combination of in-person and in-home rehabilitation); used balance-related exercises without telerehabilitation; used no objective outcome measures for evaluating balance and/or gait performance after telerehabilitation; and included no individuals with balance impairments caused by age, diseases, or clinical conditions (i.e., older adults and people with stroke, Parkinson’s disease, or multiple sclerosis).

### 2.3. Data Extraction and Tabulation 

After determining the studies included in this systematic review, both authors extracted information and data. Furthermore, both authors comprehensively examined and discussed methodologies for statistical analysis, descriptive statistics, and significant outcome measures in each study to minimize the potential confounding of the results. 

The information and data included the following: author(s); publication date; participant characteristics; sample size; balance and/or gait telerehabilitation intervention; remote monitoring and/or guidance method; exercise frequency and total duration of intervention; assessment period; and objective and/or clinical outcome assessments of balance and/or gait rehabilitation.

### 2.4. Methodological Quality

The methodological quality was assessed with the National Institute of Health’s Quality Assessment Tool for Observational Cohort and Cross-sectional Studies [21]. The tool assesses the responses “Yes”, “No”, and “Other” (“Cannot Determine”, “Not Reported”, “Not Available”) to 14 questions. Each author of this systematic review independently assessed the methodological quality of the 17 studies included. After the authors’ assessment and in-depth discussions, each study was classified into “good”, “fair”, or “low” methodological quality [21]. “Good quality” received “Yes” responses to 8 or more of the 14 questions; “fair quality” received “Yes” responses to 5, 6, or 7 questions; and “low quality” received less than 5 “Yes” responses [22].

## 3. Results

### 3.1. Literature Search

Figure 1 shows the sequential process for study selection according to PRISMA guidelines. An initial database search identified 247 studies, of which 155 were removed for duplicate records, review articles (i.e., systematic, perspective, and narrative reviews), survey articles, books and book chapters, non-English articles, and inaccessible articles. Of the 92 remaining studies, 35 were removed after title and abstract screening, and of the remaining 57, 40 were removed after full-text screening because they were not telerehabilitation or in-home training (*n* = 30); were hybrid training (i.e., in-person and in-home rehabilitation) (*n* = 1); did not provide balance or gait exercises (e.g., cardiovascular exercise) (*n* = 5); had no balance- and gait-related outcome measures (*n* = 2); and did not use individuals with balance impairments caused by age, diseases, or clinical conditions. The final number was 17 studies.

### 3.2. Study Analysis 

#### 3.2.1. Participant Characteristics

Table 1 lists the characteristics of the individuals in the 17 studies selected: older adults [23,24,25]; individuals with acute or chronic stroke [26,27,28,29,30,31]; individuals with Parkinson’s disease [32,33,34,35,36,37,38]; and individuals with multiple sclerosis [38,39]. One study with individuals with Parkinson’s disease had older adults as a control group [37], and 2 studies had no control or comparison group [25,39]. The remaining 14 studies included remote intervention and control or comparison groups and individuals with identical characteristics [23,24,26,27,28,29,30,31,32,33,34,35,36,38]. The 17 studies had sample sizes from 12 to 132.

#### 3.2.2. Study Characteristics

Table 1 lists the characteristics of the 17 studies. With the exception of 4 [25,29,34,39], 13 were either clinical or randomized controlled trials [23,24,26,27,28,30,31,32,33,35,36,37,38] with intervention and control or comparison groups. Of the 17 studies, 7, 6, and 4 used custom-built telerehabilitation systems or programs [23,24,25,26,29,34,36], commercially available or accessible videoconferencing systems [27,28,32,33,35,37], and commercially available telerehabilitation systems [30,31,38,39], respectively. 

In the 17 studies, balance and gait-related exercises included: static postural adaptation on firm and foam surfaces; dynamic postural adaptation involving the upper extremities, trunk, pelvis, hip, knees, and ankles; weight shifting; flexion-extension at shoulder, hip, knee, and ankle joints); and leg-raising, stepping, turning, making transfers involving the whole body, trunk, and upper or lower extremities (i.e., sit-to-stand, squat, walk, and dance). In total, 6 studies delivered exercises via VR [26,31,34,36,38,39], 5 delivered via smartphone or tablet apps [23,24,25,29,30], and 6 delivered via videoconferencing [27,28,32,33,35,37]. Of the 11 studies using VR, smartphone, or tablet apps, 6 used exergames [25,26,29,36,38,39]. 

The outcome measures used to assess balance and/or gait performance included 2-Minute Walk Test (2MWT); 25 Foot Walk (25FW); 30-Second Sit-to-Stand (30s S2S); Five Times Sit-to-Stand Test (5xSST); Activities-Specific Balance Confidence (ABC); Brunel Balance Assessment (BBA); Berg Balance Scale (BBS); Barthel Index (BI); Dynamic Gait Index (DGI); Fugl-Meyer Assessment (FMA); Fugl-Meyer Assessment–Balance function (FMA-Balance); Fugl-Meyer Assessment–Lower Extremity (FMA-LE); Movement Disorders Society Unified Parkinson Disease Rating Scale section for motor impairment (MDS-UPDRS III); Mini Balance Evaluations Systems Test with a total score of 28 points (Mini-BESTest28); Mini Balance Evaluations Systems Test with a total score of 32 points (Mini-BESTest32); Modified Rankin scale (mRS); Nine-Hole Pegboard Test (NHPT); Performance-Oriented Mobility Assessment balance subscale (POMA-B); Performance-Oriented Mobility Assessment gait subscale (POMA-G); Spanish version of Postural Assessment Scale for Stroke Patients (S-PASS); Spanish version of the Trunk Impairment Scale 2.0 (S-TIS 2.0); Sensory Organization Tests (SOT); Short Physical Performance Battery (SPPB); Timed Up-and-Go (TUG); Timed Up-and-Go-test Dual-task (TUG-D); and Unified Parkinson’s Disease Rating Scale (UPDRS). All 17 studies assessed balance and/or gait performance before and after telerehabilitation interventions, and 7 of the 17 studies included retention assessments ranging from 4 weeks to 6 months [24,26,27,28,33,34,36]. 

In the 17 studies, all intervention groups received telerehabilitation in conjunction with real-time or follow-up feedback and/or instructions from health professionals (i.e., physical therapists, physiotherapists, or instructors). Of the 17, 2 studies included collaborative telerehabilitation regimens involving caregivers [27] and neurologists, nurses, counsellors, and caregivers [35]. Videoconferencing was the most common telerehabilitation provider of feedback and instructions [25,27,28,30,31,32,33,34,35,37,39]. The control or comparison groups received usual care [27,35,36] or conventional rehabilitation at home [23,24,30,37,38], in a clinic [26,28,33], or in class [32]. One study included two control groups that received either cognitive therapy or cognitive and speech therapy in addition to motor exercises [31]. All 17 studies used telerehabilitation durations of 4 to 12 weeks for the intervention and control/comparison groups. Intervention groups in the 17 studies showed significant improvements in their balance and gait performance after completing telerehabilitation. Particularly, 15 studies demonstrated that intervention groups improved more than control groups or comparison groups received conventional rehabilitation (i.e., usual care, in-clinic, in-person, or in-home regimens) [23,24,26,27,28,30,32,33,35,36,37,38]. However, statistical uncertainty exists for comparisons across all 17 studies due to participant characteristics, sample size, and rehabilitation duration.

#### 3.2.3. Quality Assessment

Table 2 reports the results of the methodological quality assessment; no studies were excluded based on the results of the assessment. Since all 17 studies had objective measures, the Q12 was not applicable (N/A) and was excluded from the methodological quality assessment. All 17 studies received “Yes” responses to 12 or more of the 14 questions and were rated as good overall quality with a low risk of bias. Of the 17, 10 studies did not provide a justification or perform a power analysis for their sample sizes.

## 4. Discussion

This systematic review selected 17 studies to assess the effects of interactive telerehabilitation (VR, smartphone- and tablet-based learning apps, gamification, videoconferencing) with remote monitoring and guidance on balance and gait performance in different populations. All 17 studies included in this review demonstrated the beneficial effects of telerehabilitation for improving balance and/or gait performance in older adults, individuals with stroke, individuals with Parkinson’s disease, and individuals with multiple sclerosis. The following sub-sections discuss the contributions of the interactive telerehabilitation technologies; remote monitoring and guidance methods; and improvements in balance and gait performance for different populations. The discussion ends with the limitations of this systematic review.

### 4.1. Interactive Telerehabilitation Technologies

The target populations in all 14 studies were under care at home and performed long-term (4 weeks or longer) balance and gait training in their domestic settings (i.e., in home or in home and around home). Smartphone-based and tablet-based telerehabilitation technology particularly benefited individuals with mobility limitations and individuals living far from physical therapy facilities and clinics. The target populations used remote telerehabilitation technology to schedule their training, which allowed them to schedule sessions at their preferred time and exempted the healthcare facilities from scheduling the appointment. Potentially, telerehabilitation technology could reduce the cost of healthcare as well as the burden on healthcare facilities [40,41].

### 4.2. Data Tracking and Analysis

Over the past decade, the most commonly used methods are VR [17], training and learning apps [42], gamification [43], and videoconferencing [19]. In total, 6 of the 17 studies used VR for interactive telerehabilitation [26,31,34,36,38,39], and 5 of those 6 incorporated an off-the-shelf platform or a miniaturized motion sensor, such as Microsoft Kinect [26,36,39] and inertial sensors [31,38]. The Kinect uses cameras and depth sensors to translate body movements into a virtual environment, and the inertial sensors map the position and speed of sensor-worn body segments into a virtual object. Five of the 17 studies used smartphone or tablet-based training and learning apps for interactive telerehabilitation [23,24,25,29,30], and 4 of those 5 used inertial sensors to track the body’s motion and provided real-time biofeedback using visual or vibrotactile methods during telerehabilitation exercises [23,24,25,29]. In total, 6 of the 11 studies using VR or smartphone- or tablet-based apps included gamification (i.e., exergames) [25,26,29,36,38,39], which encourages participation and improves exercise consistency and adherence. Additionally, 6 of the 17 studies used videoconferencing (i.e., two-way video and audio technology for communicating, monitoring, and guiding individuals) [27,28,32,33,35,37]. 

Integrating VR, smartphone- or tablet-based apps, gamification, and videoconferencing into interactive telerehabilitation can improve overall accessibility, personalization, engagement, real-time monitoring and guidance, cost-effectiveness, and data-driven decision-making. Receiving sensory biofeedback while performing motor tasks can improve the individual’s acquisition of task skills and support the repetitive exercises [44,45,46]. Receiving sensory biofeedback while performing motor tasks can improve an individual’s acquisition of task skills and support the repetitive exercising [44,45,46].

### 4.3. Remote Monitoring and Guidance Methods

Remote monitoring and guidance through telecommunication technologies have increasingly been recognized as a viable approach to improve individualized care, facilitate timely intervention, empower individuals, and encourage self-management [18,19]. Several techniques have been employed for remote monitoring and guidance in telerehabilitation, including secure messaging and calling, mobile apps, videoconferencing, and healthcare platforms.

Among the 17 studies included in this review, videoconferencing was the most common method (*n* = 12). This can be primarily attributed to its relative affordability, availability, ease of use, and user-friendly freeware or commercial videoconferencing software. Of the 17 studies, 12 used videoconferencing software to administer, monitor, and supervise telerehabilitation exercises in real-time [27,28,31,32,33,34,35,37], or after the completion of exercises [25,26,30,39], and the remaining 5 used either secure messaging [23,24], phone calls [29,36], or a healthcare platform [39]. All 12 studies found that therapists or instructors supervised and monitored exercise progress; demonstrated proper exercise techniques and movements; visually assessed, guided, and adjusted body movements; adjusted exercise intensity, duration, and type; and supported and encouraged individuals during exercise regimens.

### 4.4. Limitations

The systematic review had the following limitations. First, publication bias may be present because only interactive telerehabilitation via remote monitoring and guidance described in published studies are considered. Second, using the keywords “telerehabilitation”, “remote*” or “bidirectional*” and “monitoring” or “guidance” may overlook studies published under different titles or keywords. Third, limiting the review to specific populations may prohibit generalizing the results to other populations. Fourth, using the outcome measures of balance and/or gait performance does not capture the effects of interactive telerehabilitation for cognitive, social, general well-being, and other aspects. Fifth, a language bias may be present, because only English language publications are considered. Sixth, the review cannot quantitatively compare the outcomes of the 17 studies, because of heterogeneity (i.e., demographic characteristics, telerehabilitation technology, research design, interventions, and outcome measures). Seventh, the review cannot formulate evidence-based recommendations due to the lack of a method for evaluating the quality of evidence (e.g., Grading of Recommendations Assessment, Development, and Evaluation (GRADE) [47]). Eighth, limiting the review to studies undertaken in high-income countries may prohibit generalizing the results to lower- and middle-income countries. 

## 5. Conclusions

The systematic review found strong evidence of the beneficial effects of interactive telerehabilitation via remote monitoring and guidance for balance and gait exercise regimens. The treatment outcomes of older adults and individuals with neurological conditions (i.e., stroke, Parkinson’s disease, and multiple sclerosis) were better than, or as good as, conventional in-clinic and in-home rehabilitation. The findings indicate that interactive telerehabilitation is likely to replace conventional rehabilitation methods for older adults, individuals with neurological conditions, and balance- and gait-impaired individuals of any age who cannot travel. Advances in interactive telerehabilitation technology may also promote the use of remote monitoring and guidance in a changing climate. 

The results of the review suggest the following research pursuits. Integrating artificial intelligence and machine learning into telerehabilitation technology could enhance personalization and remote interventions. The findings obtained from a widespread investigation of telerehabilitation platforms by healthcare professionals, caregivers, and target populations could improve future usability, user satisfaction, and acceptance. The cost-effectiveness and economic benefits (i.e., potential savings in healthcare resources; improvements in healthcare delivery) of telerehabilitation versus traditional rehabilitation need a comprehensive assessment. Analysis of interactive telerehabilitation implementations and secure communications infrastructure could identify the range of factors influencing the scalability and sustainability of remote interventions. An assessment of the influence of communication infrastructure on the provision and accessibility of telerehabilitation services is necessary. A study of interprofessional collaboration could reveal new methods of teamwork for healthcare professionals who provide remote monitoring and guidance and for those who provide care and assistance in the home.

## Figures and Tables

**Table 1:** Participant characteristics, intervention and training methods, details of technologies, assessment periods, and summary of outcome measures.

Study	Participant Characteristic (Sample Size)	Intervention	Remote Monitoring/Guidance Method	Balance and Gait-Related Outcome Measures	Assessment Periods	Summary of Statistically Significant Results Associated with Balance and Gait Performance (*p*-Value)
Bao et al., 2018 [23]	TRG: Older adults (*n* = 6) CG: Older adults (*n* = 6)	TRG: 6 to 8 weeks of tablet-based in-home balance and cognitive exercises (5 days per week) Progress of in-home exercise regimens monitored remotely by physical therapists In-home exercise regimens adjusted remotely by therapists CG: 6 to 8 weeks of conventional in-home balance and cognitive exercises (5 days per week) Progress of in-home exercise regimens monitored remotely by physical therapists In-home exercise regimens adjusted remotely by therapists	Email	Mini-BESTest28, Mini-BESTest32, SOT, 5xSST	Pre- and post-intervention	Both groups improved Mini-BESTest28 scores (*p* < 0.001) after in-home exercises were completed; more improvements for TRG than CG
Bao et al., 2022 [24]	TRG: Older adults (*n* = 8) CG: Older adults (*n* = 8)	TRG: 8 weeks of smartphone-based in-home balance exercises (3 days per week) Progress of in-home exercise regimens monitored remotely by physical therapists In-home exercise regimens adjusted remotely by therapists CG: 8 weeks of conventional in-home balance exercises (3 days per week) Progress of in-home exercise regimens monitored remotely by physical therapists In-home exercise regimens adjusted remotely by therapists	Email	Mini-BESTest28, SOT	Pre- and post-intervention, retention (1 month and 6 months)	Both groups improved Mini-BESTest28 and SOT scores (*p* < 0.01) after in-home exercises were completed; more improvements for TRG than CG Improved Mini-BESTest28 and SOT scores retained for 1 month and 6 months after exercises were completed regardless of group (*p* < 0.05)
Park et al., 2022 [25]	TRG: Older adults (*n* = 14)	TRG: 12 weeks of tablet-based in-home balance exergames (2 days per week) In-home exercise regimens monitored remotely by research team	Integrated video call via a tablet	Balance and mobility-related questionnaire	Pre- and post-intervention	TRG reported improved balance performance (*p* = 0.005), overall body functioning (*p* = 0.015), and physical performance (*p* = 0.015) after in-home exercises were completed
Lloréns et al., 2015 [26]	TRG: Individuals with stroke (*n* = 15) CG: Individuals with stroke (*n* = 15)	TRG: 12 weeks of VR-based in-home balance exercises using the Microsoft Kinect system (3 days per week) Progress of in-home exercise regimens monitored remotely by physical therapists CG: 12 weeks of VR-based in-clinic balance exercises (3 days per week) Progress of in-clinic exercise regimens monitored remotely by physical therapists	Face-to-face or interview	BBA, BBS, POMA-B, POMA-G	Pre- and post-intervention, retention (12 weeks)	Both groups improved balance and gait (*p* ≤ 0.02) performance after in-home exercises were completed; no difference in improved performance between TRG and CG Improved balance and gait performance retained for 12 weeks after exercises were completed regardless of group (*p* < 0.01)
van den Berg et al., 2016 [27]	TRG: Individuals with stroke (*n* = 20) CG: Individuals with stroke (*n* = 32)	TRG: 8 weeks of caregiver-mediated in-home balance exercises with support using a customized exercise app loaded onto a tablet (5 days per week) In-home exercises supervised remotely by physiotherapists CG: 12 weeks of usual care (i.e., interdisciplinary rehabilitation)	Videoconferencing via Vidyo	Extended activities of daily living, TUG	Pre- and post-intervention, retention (4 weeks)	TRG improved activities of daily living after in-home exercises were completed (*p* = 0.01); more improvements for TRG than CG Improved activities of daily living were retained for 4 weeks after exercises were completed (*p* = 0.01) CG performed better on TUG test than TRG at 4-week follow-up assessments (*p* = 0.0307)
Chen et al., 2016 [28]	TRG: Individuals with stroke (*n* = 26) CG: Individuals with stroke (*n* = 24)	TRG: 12 weeks of in-home physical exercises (5 days per week) In-home exercise regimens supervised remotely by physical therapists CG: 12 weeks of in-clinic physical exercises (5 days per week) In-clinic exercise regimens supervised by physical therapists	Videoconferencing	BI, BBS	Pre- and post-intervention, retention (12 weeks)	Both groups improved BI and BBS scores after exercises were completed (*p* < 0.001); no difference in improved performance between TRG and CG Improved balance and gait performance were retained for 12 weeks after exercises were completed regardless of group (*p* < 0.001)
Bellomo et al., 2020 [29]	TRG: Individuals with stroke (*n* = 22)	TRG: 12 weeks of in-home physiotherapy using a tablet-based exergaming system (5 days per week) Progress of in-home physiotherapy regimens monitored remotely by physiotherapists	Audio and video tutorials, phone call	BI, BBS, FMA, mRS	Pre- and post-intervention	TRG improved BI, BBS, FMA, and mRS scores after in-home exercises were completed (*p* = 0.036, *p* = 0.008, *p* = 0.003, and *p* = 0.047).
Salgueiro et al., 2022 [30]	TRG: Individuals with stroke (*n* = 15) CG: Individuals with stroke (*n* = 15)	TRG: 12 weeks of smartphone app-based in-home physiotherapy using the Farmalarm App (2 days per week) Adherence of in-home physiotherapy regimens monitored remotely by the App’s administration panel CG: 12 weeks of conventional in-home physiotherapy (2 days per week)	Face-to-face, phone or video call follow-up	BBS, S-PASS, S-TIS	Pre- and post-intervention	TRG improved balance performance (*p* = 0.02) and BBS score (*p* = 0.029) after in-home exercises were completed; more improvements for TRG than CG CG only improved BBS score (*p* = 0.009) after in-home exercises were completed While TRG improved sitting balance performance (*p* < 0.0001), CG did not improve sitting balance performance (*p* = 0.606)
Federico et al., 2023 [31]	TRG1: Individuals with stroke (*n* = 23) TRG2 (CG1): Individuals with stroke (*n* = 40) TRG3 (CG2): Individuals with stroke (*n* = 11)	TRG1-3: 4 weeks of VR-based in-home motor exercises (TRG1) or motor and cognitive exercises (TRG2), or motor, cognitive, and speech exercises (TRG3) using the Virtual Reality Rehabilitation System (VRRS) (5 days per week) In-home exercise regimens supervised remotely by physical therapists	Tablet built-in supervision with videoconferencing	BI, FMA-Balance, NHPT	Pre- and post-intervention	TRG1 and TRG2 improved BI scores (*p* = 0.021 and *p* < 0.001) Only TRG1 improved FMA-Balance scores (*p* < 0.001) TRG2 and TRG3 improved NHPT scores (*p* < 0.001 and *p* = 0.004)
Seidler et al., 2017 [32]	TRG: Individuals with Parkinson’s disease (*n* = 10) CG: Individuals with Parkinson’s disease (*n* = 10)	TRG: 12 weeks of in-home dance exercises (2 days per week) In-home exercise regimens administered remotely by instructors CG: 12 weeks of in-person dance exercises (2 days per week) In-person exercise regimens administered by instructors	Videoconferencing	MDS-UPDRS III, Mini-BESTest32	Pre- and post-intervention	Both groups improved MDS-UPDRS III and Mini-BESTest32 scores (*p* < 0.001) after in-home or in-person dance exercises were completed; no difference in improved performance between TRG and CG
Gandolfi et al., 2017 [33]	TRG: Individuals with Parkinson’s disease (*n* = 36) CG: Individuals with Parkinson’s disease (*n* = 34)	TRG: 7 weeks of VR-based in-home balance exergames using the Nintendo Wii Fit system (3 days per week) In-home exercise regimens adjusted and supervised remotely by physiotherapists CG: 7 weeks of in-clinic balance exercises (3 days per week) In-clinic exercise regimens supervised by physiotherapists	Videoconferencing via Skype	ABC, BBS, DGI	Pre- and post-intervention, retention (1 month)	Both groups improved balance and gait performance (*p* < 0.001) after in-home exercises were completed; more improvements for TRG than CG Improved balance and gait performance retained for 1 month after in-home exercises were completed regardless of group (*p* < 0.001)
Park et al., 2020 [34]	TRG: Individuals with Parkinson’s disease (*n* = 32)	TRG: 4 weeks of VR-based TeleRehabilitation with Aims to Improve Lower extremity recovery post-stroke (TRAIL) program (2 days per week) In-home TRAIL program administered remotely by physical therapists	Videoconferencing	30s S2S, FMA-LE, TUG	Pre- and Post-intervention, Retention (4 and 8 weeks)	Participants improved 30s S2S performance (*p* < 0.001), FMA-LE scores (*p* = 0.001), and TUG performance (*p* = 0.02) after in-home exercises were completed Improved balance and gait performance were retained for 4 and 8 weeks after in-home exercises were completed (*p* < 0.05)
Wu et al., 2020 [35]	TRG: Individuals with Parkinson’s disease (*n* = 30) CG: Individuals with Parkinson’s disease (*n* = 31)	TRG: 12 weeks of in-home exercises based on a collaborative care model (2 days per week) In-home exercise regimens administered remotely by collaborative team (neurologists, nurses, rehabilitation therapists, counsellors, and caregivers) CG: 12-week of routine rehabilitation and nursing measures	Videoconferencing via Internet-based TCMeeting v6.0 and phone call	BBS, FMA-LE	Pre-, Mid (4 and 8 weeks)-, and post-intervention	Both groups improved BBS and FMA-LE scores (*p* < 0.05) at 4-, 8-, 12-week assessments; more improvements for TRG than CG
Isernia et al., 2020 [36]	TRG: Individuals with Parkinson’s disease (*n* = 11) CG1: Individuals with Parkinson’s disease (*n* = 31) CG2: Individuals with Parkinson’s disease (*n* = 20)	TRG: 12 weeks of in-home Human Empowerment Aging and Disability (HEAD) rehabilitation program with VR-based exergames using the Microsoft Kinect system (5 days per week) Progress of in-home HEAD rehabilitation program remotely monitored by clinicians CG1: 4 weeks of in-clinic HEAD rehabilitation program with VR-based exergames using the Microsoft Kinect system (3 days per week) In-clinic HEAD rehabilitation program supervised by clinical professional CG2: 12-week of usual care (i.e., following recommendations of the neurologists)	Phone call	2MWT, BBS	Pre- and post-intervention, retention (3 months)	TRG improved 2 MWT performance compared to CG2 after in-home exercises were completed (*p* = 0.033); more improvements for TRG than CG2 Improved balance and gait performance retained for 3 months after in-home exercises were completed (*p* = 0.045) Compared to CG2, TRG also improved BBS scores at 3 months after in-home exercises were completed (*p* = 0.045); more improvements for TRG than CG2 CG1 improved 2MWT performance (*p* = 0.024) and BBS scores (*p* = 0.04) after in-clinic exercises were completed
Pinto et al., 2023 [37]	TRG: Individuals with Parkinson’s disease (*n* = 12) CG: Older adults (*n* = 14)	TRG: 2 months of in-home dance exercises (2 days per week) In-home exercise regimens administered remotely by certified instructors CG: 2 months of in-home dance exercises (2 days per week) In-home exercise regimens administered remotely by certified instructors	Videoconferencing via Zoom	5xSST	Pre- and post-intervention	Only TRG improved lower-limb functional mobility (*p* = 0.03) after in-home exercises were completed; more improvements for TRG than CG
Goffredo et al., 2023 [38]	TRG: Individuals with Parkinson’s disease (*n* = 35) and with multiple sclerosis (*n* = 30) CG: Individuals with Parkinson’s disease (*n* = 37) and with multiple sclerosis (*n* = 30)	TRG: 6 to 8 weeks of VR-based in-home balance and cognitive exercises using the Virtual Reality Rehabilitation System (VRRS) (5 days per week) In-home exercise regimens performed with a virtual assistant CG: 6 to 8 weeks of conventional in-home balance and cognitive exercises (5 days per week) In-home exercise regimens performed with a self-administered booklet	Therapist’s pre-determined exercise guidance corresponded to participants’ characteristics and needs	Mini-BESTest28, TUG, TUG-D	Pre- and post-intervention	Both groups improved Mini-BESTest28 (*p* < 0.001) after in-home exercises were completed; more improvements for TRG than CG Both groups improved timed up-and-go performance with (*p* = 0.002) and without dual task (*p* < 0.001) after in-home exercises were completed; more improvements for TRG than CG
Chanpimol et al., 2020 [39]	TRG: Individuals with multiple sclerosis (*n* = 10)	TRG: 12 weeks of VR-based in-home balance exergames using Jintronix^®^ (3 or more days per week) In-home exercise regimens remoted adjusted by therapists Clinical video teleconferencing follow-ups	Teleconferencing via a secure web-based portal	2MWT, 25FW, SPPB		TRG improved ambulation speed (*p* = 0.04), distance (*p* = 0.002), and SPPB score (*p* = 0.04) after in-home exercises were completed

Notes: TRG: Telerehabilitation group; CG: control/comparison group; 2MWT: 2-Minute Walk Test; 25FW: 25 Foot Walk; 30s S2S: 30-Second Sit-to-Stand; 5xSST: Five Times Sit to Stand Test; ABC: Activities-Specific Balance Confidence; BBA: Brunel Balance Assessment; BBS: Berg Balance Scale; BI: Barthel Index; DGI: Dynamic Gait Index; FMA: Fugl-Meyer Assessment; FMA-Balance: Fugl-Meyer Assessment–Balance function; FMA-LE: Fugl-Meyer Assessment–Lower Extremity; MDS-UPDRS III: Movement Disorders Society Unified Parkinson Disease Rating Scale section for motor impairment; Mini-BESTest28: Mini Balance Evaluations Systems Test (a total score of 28 points); Mini-BESTest32: Mini Balance Evaluations Systems Test (a total score of 32 points); mRS: Modified Rankin scale; NHPT: Nine-Hole Pegboard Test; POMA-B: Performance-Oriented Mobility Assessment balance subscale; POMA-G Performance-Oriented Mobility Assessment gait subscale; S-PASS: Spanish version of Postural Assessment Scale for Stroke Patients; S-TIS 2.0: Spanish version of the Trunk Impairment Scale 2.0; SOT: Sensory Organization Tests; SPPB: Short Physical Performance Battery; TUG: Timed Up-and-Go; TUG-D: Timed Up-and-Go-test Dual-task; UPDRS: Unified Parkinson’s Disease Rating Scale; and VR: Virtual Reality.

**Table 2:** Results of the methodological quality assessment.

Study	Q1	Q2	Q3	Q4	Q5	Q6	Q7	Q8	Q9	Q10	Q11	Q12	Q13	Q14	Overall
Bao et al., 2018 [23]	Yes	Yes	Yes	Yes	Yes	Yes	Yes	Yes	Yes	Yes	Yes	N/A	Yes	Yes	Good
Bao et al., 2022 [24]	Yes	Yes	Yes	Yes	No	Yes	Yes	Yes	Yes	Yes	Yes	N/A	Yes	Yes	Good
Park et al., 2022 [25]	Yes	Yes	Yes	Yes	Yes	Yes	Yes	Yes	Yes	Yes	Yes	N/A	Yes	Yes	Good
Lloréns et al., 2015 [26]	Yes	Yes	Yes	Yes	Yes	Yes	Yes	Yes	Yes	Yes	Yes	N/A	Yes	Yes	Good
van den Berg et al., 2016 [27]	Yes	Yes	Yes	Yes	No	Yes	Yes	Yes	Yes	Yes	Yes	N/A	Yes	Yes	Good
Chen et al., 2016 [28]	Yes	Yes	Yes	Yes	No	Yes	Yes	Yes	Yes	Yes	Yes	N/A	Yes	Yes	Good
Bellomo et al., 2020 [29]	Yes	Yes	Yes	Yes	Yes	Yes	Yes	Yes	Yes	Yes	Yes	N/A	Yes	Yes	Good
Salgueiro et al., 2022 [30]	Yes	Yes	Yes	Yes	No	Yes	Yes	Yes	Yes	Yes	Yes	N/A	Yes	Yes	Good
Federico et al., 2023 [31]	Yes	Yes	Yes	Yes	No	Yes	Yes	Yes	Yes	Yes	Yes	N/A	Yes	Yes	Good
Seidler et al., 2017 [32]	Yes	Yes	Yes	Yes	No	Yes	Yes	Yes	Yes	Yes	Yes	N/A	Yes	Yes	Good
Gandolfi et al., 2017 [33]	Yes	Yes	Yes	Yes	No	Yes	Yes	Yes	Yes	Yes	Yes	N/A	Yes	Yes	Good
Park et al., 2020 [34]	Yes	Yes	Yes	Yes	Yes	Yes	Yes	Yes	Yes	Yes	Yes	N/A	Yes	Yes	Good
Wu et al., 2020 [35]	Yes	Yes	Yes	Yes	Yes	Yes	Yes	Yes	Yes	Yes	Yes	N/A	Yes	Yes	Good
Isernia et al., 2020 [36]	Yes	Yes	Yes	Yes	Yes	Yes	Yes	Yes	Yes	Yes	Yes	N/A	Yes	Yes	Good
Pinto et al., 2023 [37]	Yes	Yes	Yes	Yes	No	Yes	Yes	Yes	Yes	Yes	Yes	N/A	Yes	Yes	Good
Goffredo et al., 2023 [38]	Yes	Yes	Yes	Yes	No	Yes	Yes	Yes	Yes	Yes	Yes	N/A	Yes	Yes	Good
Chanpimol et al., 2020 [39]	Yes	Yes	Yes	Yes	No	Yes	Yes	Yes	Yes	Yes	Yes	N/A	Yes	Yes	Good

Note: Q1: Was the research question or objective in this paper clearly stated?; Q2: Was the study population specified and defined?; Q3: Was the participation rate of eligible persons at least 50%?; Q4: Were all the subjects selected or recruited from the same or similar populations (including the same time period) and were inclusion and exclusion criteria for being in the study prespecified and applied uniformly to all participants?; Q5: Was a sample size justification, power description, or variance and effect estimates provided?; Q6: For the analyses in this paper, were the exposure(s) of interest measured prior to the outcome(s) being measured?; Q7: Was the timeframe sufficient so that one could reasonably expect to see an association between exposure and outcome if it existed?; Q8: For exposures that can vary in amount or level, did the study examine different levels of the exposure as related to the outcome (e.g., categories of exposure, or exposure measured as continuous variable)?; Q9: Were the exposure measures (independent variables) clearly defined, valid, reliable, and implemented consistently across all study participants?; Q10: Was the exposure(s) assessed more than once over time?; Q11: Were the outcome measures (de-pendent variables) clearly defined, valid, reliable, and implemented consistently across all study participants?; Q12: Were the outcome assessors blinded to the exposure status of participants?; Q13: Was loss to follow-up after baseline 20% or less?; and Q14: Were key potential confounding variables measured and adjusted statistically for their impact on the relationship between exposure(s) and outcome(s)?

## Data Availability

Data is available in the manuscript.
